# Multifarious Characterization and Efficacy of Three Phosphate-Solubilizing *Aspergillus* Species as Biostimulants in Improving Root Induction of Cassava and Sugarcane Stem Cuttings

**DOI:** 10.3390/plants12203630

**Published:** 2023-10-20

**Authors:** Surapong Khuna, Jaturong Kumla, Sirasit Srinuanpan, Saisamorn Lumyong, Nakarin Suwannarach

**Affiliations:** 1 Center of Excellence in Microbial Diversity and Sustainable Utilization, Chiang Mai University, Chiang Mai 50200, Thailand; surapongway@gmail.com (S.K.); jaturong_yai@hotmail.com (J.K.); sirasit.s@cmu.ac.th (S.S.); saisamorn.l@cmu.ac.th (S.L.); 2 Office of Research Administration, Chiang Mai University, Chiang Mai 50200, Thailand; 3 Department of Biology, Faculty of Science, Chiang Mai University, Chiang Mai 50200, Thailand; 4 Academy of Science, The Royal Society of Thailand, Bangkok 10300, Thailand

**Keywords:** economic plants, fungal inoculant, phosphate solubilization, plant growth promotion, soil fungi

## Abstract

Several soil fungi significantly contribute to the enhancement of plant development by improving nutrient uptake and producing growth-promoting metabolites. In the present study, three strains of phosphate-solubilizing fungi, namely, *Aspergillus chiangmaiensis* SDBR-CMUI4, *A. pseudopiperis* SDBR-CMUI1, and *A. pseudotubingensis* SDBR-CMUO2, were examined for their plant-growth-promoting capabilities. The findings demonstrated that all fungi showed positive siderophore production, but only *A. pseudopiperis* can produce indole-3-acetic acid. All fungi were able to solubilize insoluble phosphate minerals [Ca_3_(PO_4_)_2_ and FePO_4_] by producing phosphatase enzymes and organic acids (oxalic, tartaric, and succinic acids). These three fungal species were grown at a water activity ranging from 0.837 to 0.998, pH values ranging from 4 to 9, temperatures between 4 and 40 °C, and 16–17% NaCl in order to evaluate their drought, pH, temperature, and salt tolerances, respectively. Moreover, the results indicated that *A. pseudopiperis* and *A. pseudotubingensis* were able to tolerate commercial insecticides (methomyl and propargite) at the recommended dosages for field application. The viability of each fungal strain in the inoculum was higher than 50% at 4 and 20 °C after 3 months of storage. Subsequently, all fungi were characterized as plant-growth-promoting strains by improving the root inductions of cassava (*Manihot esculenta* Crantz) and sugarcane (*Saccharum officinarum* L.) stem cuttings in greenhouse experiments. No symptoms of plant disease were observed with any of the treatments involving fungal inoculation and control. The cassava and sugarcane stem cuttings inoculated with fungal strains and supplemented with Ca_3_(PO_4_)_2_ exhibited significantly increased root lengths, shoot and root dry biomasses, chlorophyll concentrations, and cellular inorganic phosphate contents. Therefore, the application of these phosphate-solubilizing fungi is regarded as a new frontier in the induction of roots and the promotion of growth in plants.

## 1. Introduction

The availability of micro- and macro-elements in agricultural soils has a significant impact on the growth, development, and production of plants [1]. Phosphorus (P) is one of the most important macro-elements and is required for almost all of the major metabolic processes in plants, such as signal transduction, energy transfer reactions, respiration, macromolecular biosynthesis, and photosynthesis [2,3,4]. It is extensively distributed in soils in both inorganic/insoluble (containing aluminum, calcium, and iron phosphates) and organic (organic matter) forms [5,6]. Many agricultural soils contain a substantial total P in the range of 500 to 800 mg/kg of dry soil [7]. However, only 0.1% of the total P in the soil has been shown to be available for assimilation by plants [3,8,9]. Soils are frequently deficient in phosphorus because the majority of the element is insoluble and inaccessible to plants [10]. Consequently, chemical-based fertilizers are widely utilized in order to increase the concentration of available elements, especially P, in the soil that are accessible for the growth and production of crops [2]. Unfortunately, using chemical-based fertilizers excessively, frequently, and inappropriately has resulted in a number of negative side effects, including a decrease in soil fertility, a reduction in microbial diversity, environmental degradation, a decrease in crop yield, and an increase in crop production costs, and this has also caused harm to the health of farmers and consumers [5,11]. Therefore, the concept of employing phosphate-solubilizing fungi as biostimulant suppliers for readily available soil phosphorus for plants provides a strategically advantageous approach from both an economic and environmental perspective [3,12].

Phosphate-solubilizing fungi (PSF) are a type of advantageous microorganism that have the ability to process insoluble forms of phosphorus into soluble forms that can be assimilated easily by plants [13,14]. These fungi are widely dispersed throughout the soil and frequently related to plant rhizospheres [15]. Earlier research suggests that PSF constitutes approximately 0.1% to 0.5% of the overall fungal populations in soil [3,16,17]. These fungi are able to dissolve insoluble phosphate minerals due to their ability to lower pH levels by secreting organic acids (e.g., citric, gluconic, malic, oxalic, succinic, and tartaric acids) and via chelation, exchange reactions, and mineralization [4,18,19,20,21]. In addition to making soluble phosphorus available for plant uptake, PSF can also enhance plant growth, induce systematic resistance, and increase stress tolerance through one of the following mechanisms: the production of phytohormones, antibiotics, and siderophores; the solubilization of various other insoluble minerals (calcium, copper, cobalt, iron, manganese, magnesium, potassium, and zinc); and biocontrol activity [3,22,23,24]. Numerous prior studies have demonstrated that the inoculation of PSF belonging to the genera *Aspergillus*, *Penicillium*, *Rhizopus*, *Talaromyces*, and *Trichoderma* could enhance root and plant growth for several plants (Arabidopsis, chickpea, chili, haricot beans, maize, mung beans, onions, soybeans, and tomatoes) while also improving soil fertility [23,25,26,27,28,29,30,31,32,33].

From our previous study, three *Aspergillus* strains, including *Aspergillus chiangmaiensis* SDBR-CMUI4, *A. pseudopiperis* SDBR-CMUI1, and *A. pseudotubingensis* SDBR-CMUO2, can solubilize Ca_3_(PO_4_)_2_ and promote the growth of Arabidopsis [*Arabidopsis thaliana* (L.) Heynh] and onion (*Allium cepa* L.) [23]. However, their other plant-growth-promoting abilities and tolerances to drought, pH, temperature, salinity, and agrochemicals have not yet been determined. Therefore, the objective of this research was to investigate their plant-growth-promoting abilities, including the production of indole-3-acetic acid (IAA) and siderophore. The production of phosphatase enzymes and organic acids for the solubilization of different forms of phosphate minerals (AlPO_4_, Ca_3_(PO_4_)_2_, and FePO_4_) was determined. Subsequently, the capacity of these fungi to tolerate conditions of drought, pH values, temperatures, salinity, and the presence of agrochemicals was examined. The viability of fungi in a granular inoculum was studied. Furthermore, the fungal strains were evaluated in terms of their ability to improve root induction in cassava and sugarcane stem cuttings in a greenhouse experiment. The findings obtained from this study will be utilized for the development of a biostimulant-based inoculum using phosphate-solubilizing *Aspergillus*, potentially serving as an alternative to chemical-based fertilizers and supporting sustainable agricultural practices in the future.

## 2. Results

### 2.1. Characterization of Plant Growth Promotion Properties

#### 2.1.1. Determination of IAA Production

After one week, only the culture supernatant from *A. pseudopiperis* exhibited a positive reaction for IAA, as evidenced by the development of a red to pink color when tested using Salkowski’s reagent. A negative result for IAA detection was obtained in the uninoculated medium and culture supernatants from both *A. chiangmaiensis* and *A. pseudotubingensis*. Subsequently, the presence of IAA was verified through high-performance liquid chromatography (HPLC). The examination revealed the existence of IAA generated by *A. pseudopiperis*, aligning with the authentic IAA standard that exhibited a retention time of 10.1 min and a peak absorption at 279 nm. Additionally, the identity of fungal IAA was confirmed by a co-injection with the corresponding reference standard. HPLC was also used to measure the concentration of fungal IAA. It was found that *A. pseudopiperis* produced IAA at a level of 33.37 µg/mL (Table 1).

#### 2.1.2. Determination of Siderophore Production

**Figure 1:**
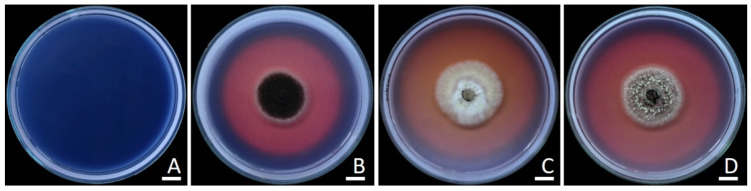
The alteration in color zones on chrome azurol S (CAS) agar due to siderophore production in uninoculated medium (**A**), *A. chiangmaiensis* SDBR-CMUI4 (**B**), *A. pseudopiperis* SDBR-CMUI1 (**C**), and *A. pseudotubingensis* SDBR-CMUO2 (**D**). Scale bars: 10 mm.

The findings revealed that all fungal strains exhibited positive siderophore production, which was evident through the formation of a pink zone around their colonies (Table 1 and Figure 1).

#### 2.1.3. Solubilization of Phosphate Minerals, Phosphatase Activities, and Organic Acid Production

**Figure 2:**
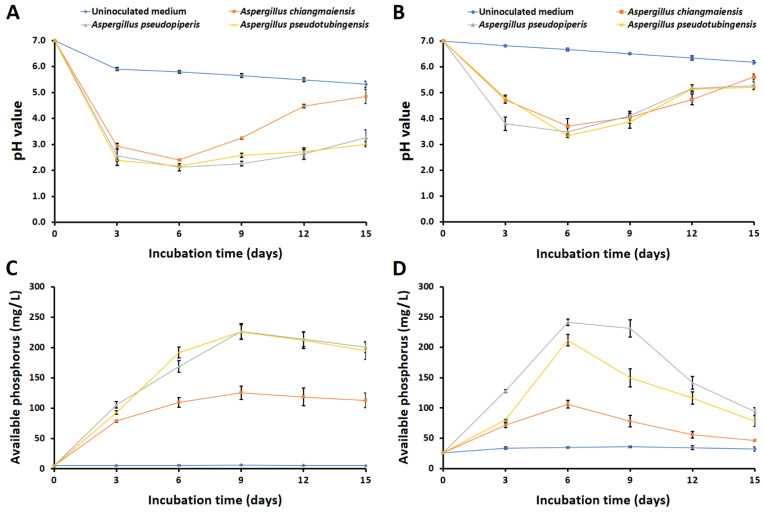
Changes in pH and available phosphorus concentration over time during solubilizations of Ca_3_(PO_4_)_2_ (**A**,**C**) and FePO_4_ (**B**,**D**).

All fungi could no longer grow in the liquid medium supplemented with AlPO_4_. During the 15 days of cultivation, the pH values of the culture supernatants from the liquid medium supplemented with Ca_3_(PO_4_)_2_ and FePO_4_ significantly decreased compared to the culture supernatants from the uninoculated treatments (Figure 2A,B). The pH values of the culture supernatants in the solubilizations of Ca_3_(PO_4_)_2_ and FePO_4_ were observed within the ranges of 3.34–5.61 and 2.12–4.84, respectively. The lowest pH values were observed six days after incubation in the solubilizations of both Ca_3_(PO_4_)_2_ and FePO_4_. For the solubilization of Ca_3_(PO_4_)_2_, *A. pseudotubingensis* displayed the lowest pH value (pH 3.34), followed by *A. pseudopiperis* (pH 3.49) and *A. chiangmaiensis* (pH 3.72) (Figure 2A). Additionally, the lowest pH value in the FePO_4_ solubilization was recorded for *A. pseudopiperis* (pH 2.12), followed by *A. pseudotubingensis* (pH 2.16) and *A. chiangmaiensis* (pH 2.40) (Figure 2B). However, the pH values started increasing accordingly with the slight increase in the incubation time.

During the 15 days of cultivation, the amount of available phosphorus solubilized by the fungi was measured from culture supernatants. The results indicated that the amount of available phosphorus solubilized by each fungal strain was significantly higher than that solubilized by the uninoculated treatments (Figure 2C,D). After 15 days of incubation, the content of available phosphorus from the experiments evaluating Ca_3_(PO_4_)_2_ and FePO_4_ solubilizations ranged from 78.79 to 226.34 mg/L and from 46.50 to 241.48 mg/L, respectively. The maximum available phosphorus of each fungal strain was present in the medium supplemented with Ca_3_(PO_4_)_2_ and FePO_4_ after being incubated for nine and six days, respectively. In terms of the solubilization of Ca_3_(PO_4_)_2_, *A. pseudopiperis* had the highest available phosphorus content (226.34 mg/L), followed by *A. pseudotubingensis* (225.78 mg/L) and *A. chiangmaiensis* (125.45 mg/L) after nine days of incubation (Figure 2C). In addition, the highest available phosphorus content was found with *A. pseudopiperis* (241.48 mg/L), followed by *A. pseudotubingensis* (211.73 mg/L) and *A. chiangmaiensis* (106.13 mg/L) after six days of incubation in the solubilization of FePO_4_ (Figure 2D).

**Figure 3:**
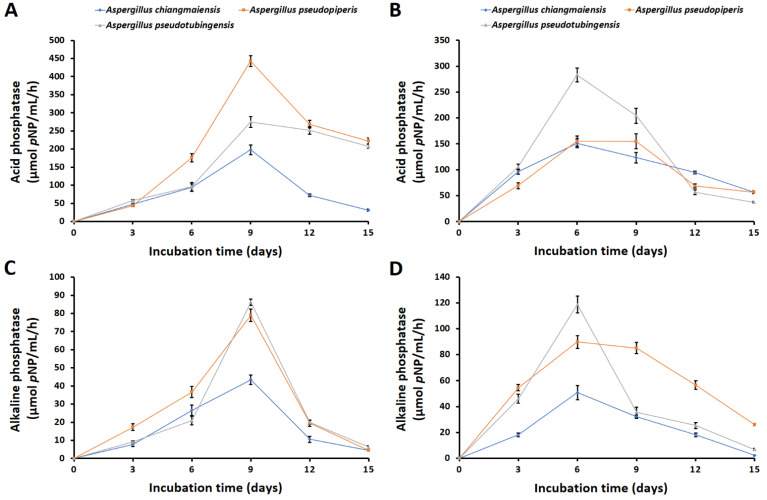
Acid and alkaline phosphatase activities of phosphate-solubilizing fungi during solubilizations of Ca_3_(PO_4_)_2_ (**A**,**C**) and FePO_4_ (**B**,**D**).

During the solubilizations of Ca_3_(PO_4_)_2_ and FePO_4_, three fungi were found to produce both acid and alkaline phosphatases during the solubilizations of Ca_3_(PO_4_)_2_ and FePO_4_ in the liquid medium. It was found that the acid phosphatase activity was higher than that for the alkaline phosphatase (Figure 3). The values of acid phosphatases in the liquid medium during the solubilizations of Ca_3_(PO_4_)_2_ and FePO_4_ were within the ranges of 31.24–442.67 and 37.14–283.33 µmol *p*NP/mL/h, respectively (Figure 3A,B). *Aspergillus pseudopiperis* showed the highest activity of acid phosphatase in the presence of Ca_3_(PO_4_) (442.67 µmol *p*NP/mL/h), followed by *A. pseudotubingensis* (274.95 µmol *p*NP/mL/h) and *A. chiangmaiensis* (197.71 µmol *p*NP/mL/h) at day 9 of incubation, respectively (Figure 3A). Furthermore, the highest acid phosphatase in the presence of FePO_4_ was found with *A. pseudotubingensis* (283.33 µmol *p*NP/mL/h), followed by *A. pseudopiperis* (155.24 µmol *p*NP/mL/h) and *A. chiangmaiensis* (151.33 µmol *p*NP/mL/h) at day 6 of incubation, respectively (Figure 3B).

The quantities of alkaline phosphatases in the medium during the solubilizations of Ca_3_(PO_4_)_2_ and FePO_4_ were within the ranges 4.49–86.26 and 2.22–118.78 µmol *p*NP/mL/h, respectively (Figure 3C,D). The highest activity of alkaline phosphatase in the medium supplemented with Ca_3_(PO_4_)_2_ was observed with *A. pseudotubingensis* (86.26 µmol *p*NP/mL/h), followed by *A. pseudopiperis* (78.98 µmol *p*NP/mL/h) and *A. chiangmaiensis* (43.46 µmol *p*NP/mL/h) at day 9 of incubation (Figure 3A). However, the highest alkaline phosphatase in the medium supplemented with FePO_4_ was obtained with *A. pseudotubingensis* (118.78 µmol *p*NP/mL/h), followed by *A. pseudopiperis* (78.98 µmol *p*NP/mL/h) and *A. chiangmaiensis* (89.75 and 50.80 µmol *p*NP/mL/h) at day 6 of incubation (Figure 3B). The amounts of acid and alkaline phosphatases produced by all fungal strains were found to be correlated with an increase in the amount of available phosphorus. However, the results of the acid and alkaline phosphatase activities were both negative in the uninoculated medium.

**Figure 4:**
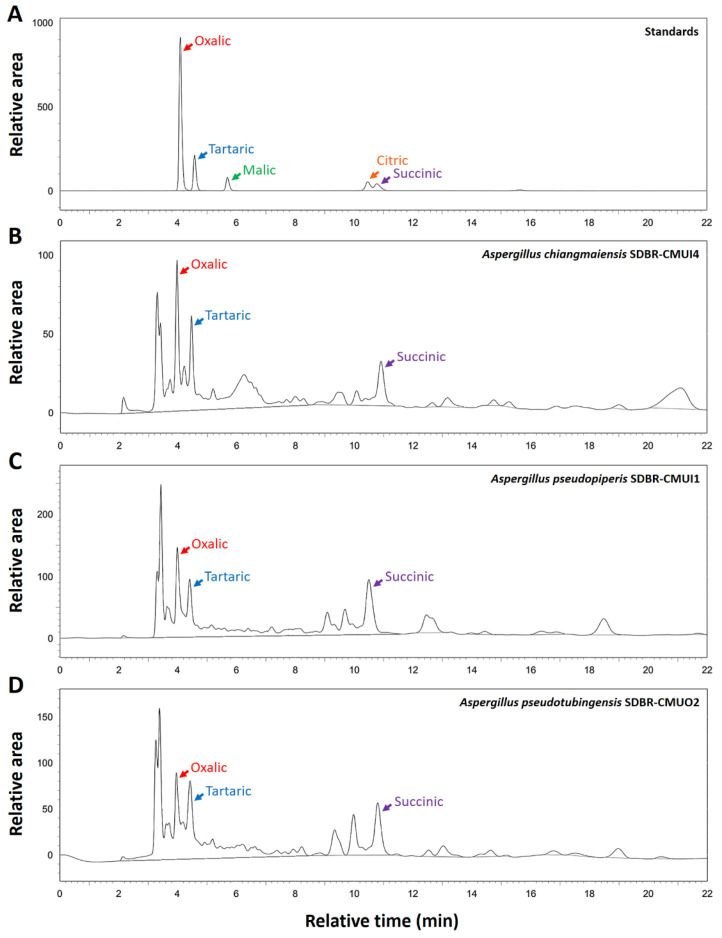
HPLC chromatograms of culture supernatant in solubilization of Ca_3_(PO_4_)_2_. Organic acid standard (**A**), A. *chiangmaiensis* SDBR-CMUI4 (**B**), A. *pseudopiperis* SDBR-CMUI1 (**C**), and A. *pseudotubingensis* SDBR-CMUO2 (**D**).

The organic acid production from fungi after 15 days of Ca_3_(PO_4_)_2_ and FePO_4_ solubilizations was determined via HPLC. The findings revealed that the organic acid content in the culture supernatants corresponded to the organic acid standards of oxalic acid, tartaric acid, and succinic acid with a relation time of 4.0, 4.5, and 10.7 min, respectively (Figure 4).

**Figure 5:**
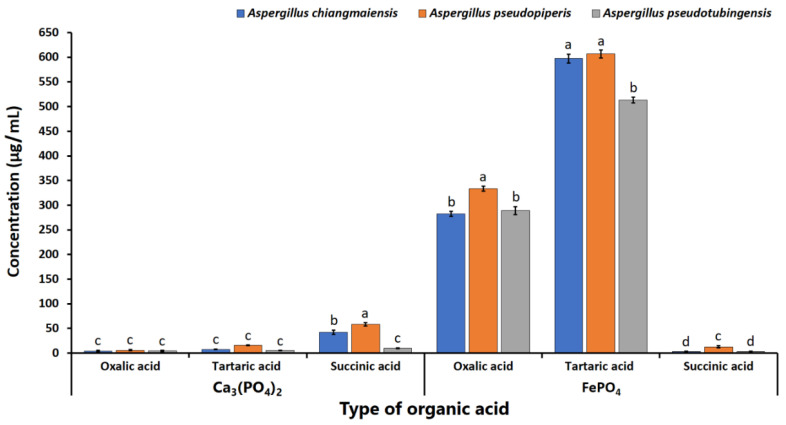
Production of organic acids after 15 days of the process of solubilization of Ca_3_(PO_4_)_2_ and FePO_4_ in a liquid medium. The error bars indicate the standard deviation of the average. Distinct letters denote a statistically significant difference (*p* ≤ 0.05).

The concentration of organic acid in the culture supernatants was also assessed using HPLC and is shown in Figure 5. The obtained values of oxalic acid, tartaric acid, and succinic acid contents of all strains were within the ranges of 4.16–333.65, 5.21–606.88, and 2.79–58.66 µg/mL, respectively. In terms of the solubilization of FePO_4_, tartaric acid was dominant, followed by oxalic acid and succinic acid. The highest concentrations of tartaric and oxalic acids were observed in the culture supernatants from the liquid medium supplemented with FePO_4_ and inoculated with each phosphate-solubilizing fungus. Additionally, all strains produced the highest concentration of succinic acid in the presence of Ca_3_(PO_4_)_2_. However, organic acids were not detected in the uninoculated treatments.

### 2.2. Drought, pH, Temperature, and Salinity Tolerances

Under conditions of drought, all fungal species were capable of growth. Both *A. chiangmaiensis* and *A. pseudotubingensis* demonstrated exceptional drought resistance in vitro, successfully growing in agar with a water availability (a_w_) of 0.837. However, *A. pseudopiperis* could be grown in up to an a_w_ value of 0.859. All fungal strains were able to grow on agar within the pH range of 4.0 to 9.0 but did not grow at a pH of 3.0. Additionally, it was found that all fungi could grow at temperatures ranging from 4 to 40 °C. Moreover, *A. chiangmaiensis* and *A. pseudotubingensis* exhibited a salinity tolerance at up to 17% NaCl, whereas *A. pseudopiperis* displayed tolerance to salinity up to 16% NaCl (Table 1).

### 2.3. Agrochemical Tolerance

**Figure 6:**
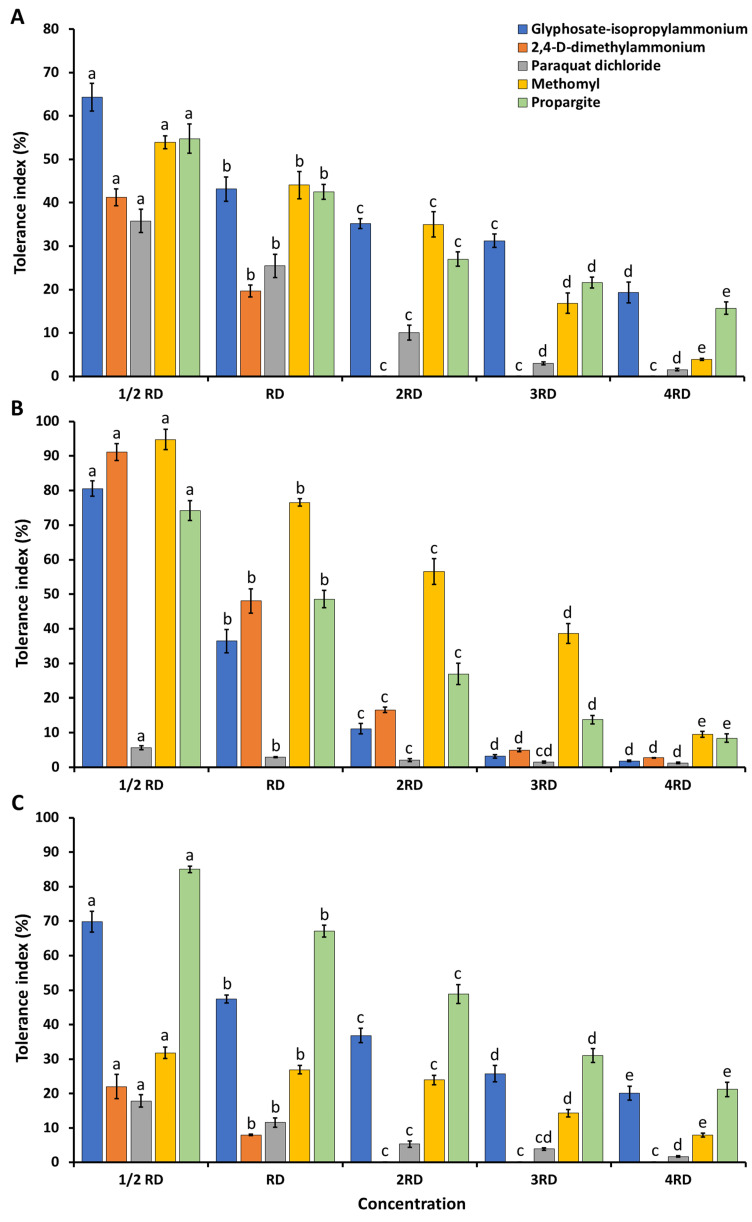
Assessment of the tolerance index of three phosphate-solubilizing fungi at various doses of five agrochemicals. *Aspergillus chiangmaiensis* SDBR-CMUI4 (**A**); *A. pseudopiperis* SDBR-CMUI1 (**B**); *A. pseudotubingensis* SDBR-CMUO2 (**C**). The error bars indicate the standard deviation of the average. Distinct letters in each experiment denote a statistically significant difference (*p* ≤ 0.05); 1/2 RD, RD, 2RD, 3RD, and 4RD indicate half of the recommended dosage, recommended dosage, double the recommended dosage, triple the recommended dosage, and quadruple the recommended dosage, respectively.

Three herbicides (glyphosate-isopropylammonium, 2,4-D-dimethylammonium, and paraquat dichloride), two fungicides (metalaxyl and benomyl), and two insecticides (methomyl and propargite) were used in this study. The tolerance of all strains to these agrochemicals is expressed as the TI value, which is shown in Figure 6. The TI value was found to decrease when the concentration of all agrochemical chemicals increased. All of the tested fungicide compounds showed a strong inhibition of the growth of all of the tested fungal species. The outcome showed that paraquat dichloride inhibited the growth of all fungal species (TI value < 50%) at all tested dosages. All if the fungal species were tolerant (TI value > 50%) to glyphosate-isopropylammonium and propargite at half of the recommended dosages. *Aspergillus chiangmaiensis* could tolerate methomyl at half of the recommended dosage (1/2 RD) (Figure 6A). Interestingly, *A. pseudopiperis* showed the highest growth tolerance at half of the recommended dosage of 2,4-D-dimethylammonium and at the recommended dosage (RD) and double the recommended (2RD) dosage of methomyl (Figure 6B). Moreover, *A. pseudotubingensis* could tolerate propargite at the recommended dosages (Figure 6C).

### 2.4. Evaluation of Fungal Viability in a Granular Inoculum

**Figure 7:**
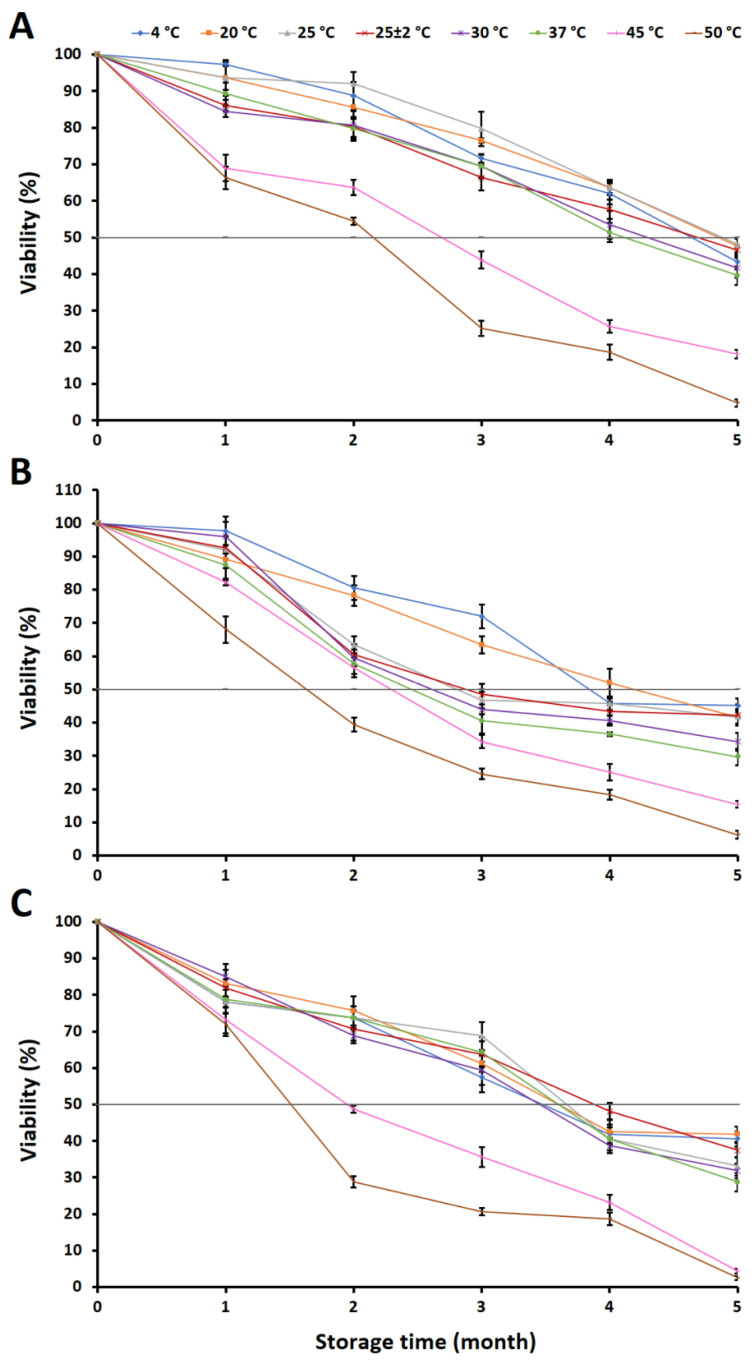
Viability of the phosphate-solubilizing fungi in granular inoculum during storage at different temperatures for five months. *Aspergillus chiangmaiensis* SDBR-CMUI4 (**A**); *A. pseudopiperis* SDBR-CMUI1 (**B**); *A. pseudotubingensis* SDBR-CMUO2 (**C**).

The viability of each fungus in each granular inoculum was determined, and the results are shown in Figure 7. It was found that the viability of each fungus decreased with the increasing storage time and was significantly affected by the storage temperature.

The viability of the fungi was reduced to approximately 50% after 3 to 4 months of storage. Among the different storage temperatures, the granular inoculum containing *A. chiangmaiensis* stored at 25 °C retained the maximum viability at 79.68%, followed by 20 °C (76.47%) and 4 °C (71.66%) after 3 months of storage. The minimum viability was observed in the granular inoculum that was stored at 50 °C (25.14%). Nevertheless, the granular inoculum that was stored at 4–37 °C for 4 months showed a viability of this fungus of higher than 50% within the range of 51.34–63.64%. The viability of *A. chiangmaiensis* was reduced to lower than 50% after storage for 5 months at all temperatures (Figure 7A).

The granular inoculum containing *A. pseudopiperis* stored at 4 °C retained the maximum viability (72.00%); this was followed by 20 °C (63.43%) after 3 months of storage. The minimum viability was observed in the granular inoculum that was stored at 50 °C (25.14%). However, the granular inoculum that was stored at 20 °C for 4 months showed the viability of this fungus as being higher than 50% (52.00%). The viability of *A. pseudopiperis* was reduced to less than 50% after storage for 5 months at all temperatures (Figure 7B).

The granular inoculum containing *A. pseudotubingensis* stored at 25 °C retained the maximum viability (68.75%), followed by 37 °C (64.38%) and room temperature (63.75%) after 3 months of storage. However, the minimum viability was observed in the granular inoculum stored at 50 °C (20.63%). The viability of this fungus was reduced to less than 50% after storage for 4 months at all temperatures (Figure 7C).

### 2.5. Root Induction of Cassava and Sugarcane Stem Cuttings under Greenhouse Conditions

#### 2.5.1. Cassava Stem Cuttings

**Figure 8:**
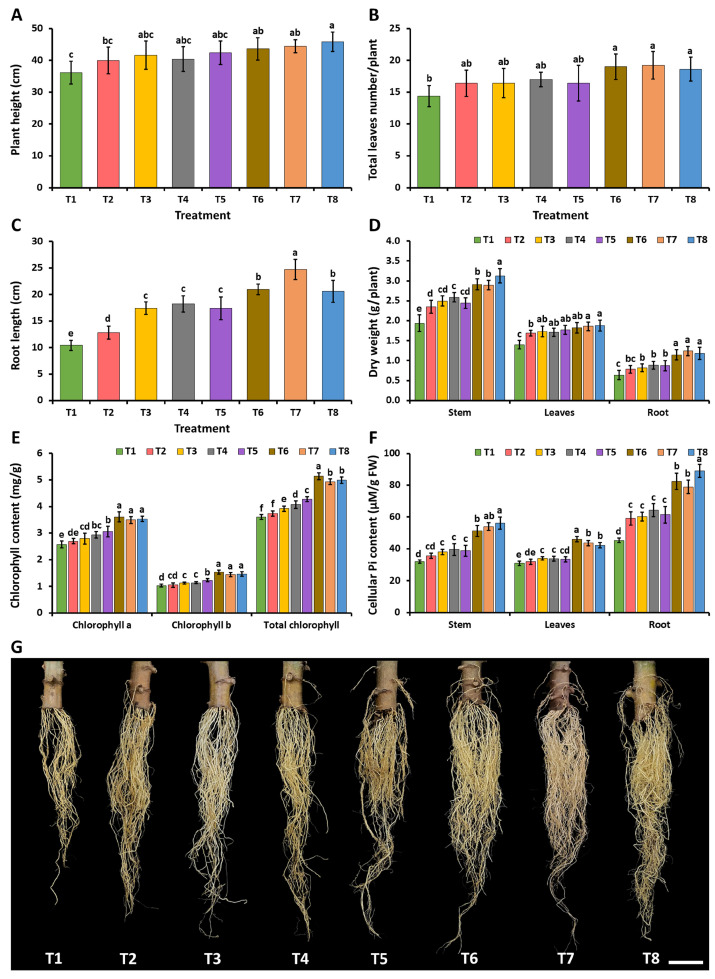
Effect of phosphate-solubilizing fungi on the growth and root induction of cassava stem cuttings. Plant height (**A**); total leaf number (**B**); root length (**C**); dry weights of stems, leaves, and roots (**D**); chlorophyll content (**E**); cellular inorganic phosphate content (**F**); root of cassava in each treatment (**G**). The error bars indicate the standard deviation of the average. Distinct letters in each experiment denote a statistically significant difference (*p* ≤ 0.05). Scale bar: (**G**) 3 cm.

During the planting period, neither the fungal inoculation treatment nor the controls exhibited any indication of plant disease. At 60 days after planting, the findings demonstrate that the cassava stem cuttings inoculated with each fungus along with the addition of Ca_3_(PO_4_)_2_ (T6–T8) had significantly higher plant heights, numbers of leaves, root lengths, and dried weights of the stems, leaves, and roots than the plants undergoing the control treatment (T1) (Figure 8A–D,G). Regarding the addition of Ca_3_(PO_4_)_2_ and inoculation with *A. pseudopiperis* (T7), the experiment showed a significantly greater root length than the inoculations with *A. chiangmaiensis* (T6) and *A. pseudotubingensis* (T8). This outcome could potentially be attributed to the production of IAA by *A. pseudopiperis*. Additionally, it was observed that the cassava stem cuttings inoculated with each fungus (T3–T5) showed longer roots and higher dry weights for the stems, leaves, and roots when compared to the plants receiving the control treatment (T1).

The concentrations of chlorophyll and cellular inorganic phosphate in the leaves, stems, and roots of the cassava stem cuttings are represented in Figure 8E, and Figure 8F, respectively. The outcomes demonstrated that the inoculation of each fungus in both treatments without additional Ca_3_(PO_4_)_2_ (T3–T5) or with additional Ca_3_(PO_4_)_2_ (T6–T8) significantly increased the chlorophyll content in the leaves and the cellular inorganic phosphate content in the leaves, stems, and roots of the cassava stem cuttings when compared to the control treatment (T1).

#### 2.5.2. Sugarcane Stem Cuttings

**Figure 9:**
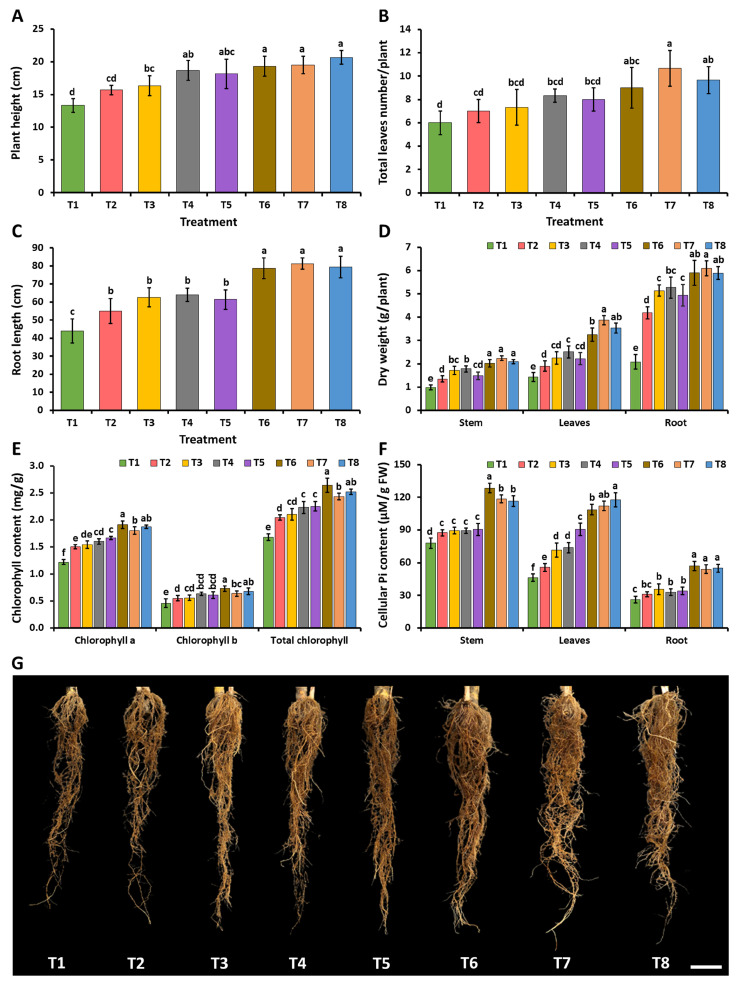
Effect of phosphate-solubilizing fungi on the growth and root induction of sugarcane stem cuttings. Plant height (**A**); total leaf number (**B**); root length (**C**); dry weights of stems, leaves, and roots (**D**); chlorophyll content (**E**); cellular inorganic phosphate content (**F**); root of sugarcane in each treatment (**G**). The error bars indicate the standard deviation of the average. Distinct letters in each experiment denote a statistically significant difference (*p* ≤ 0.05). Scale bar: (**G**) 10 cm.

None of the treatments for fungal inoculation and the control exhibited indications of plant disease throughout the planting period of 60 days. The outcomes found that the inoculation of each fungus in both treatments without additional Ca_3_(PO_4_)_2_ (T3–T5) or with additional Ca_3_(PO_4_)_2_ (T6–T8) could enhance the plant heights, root lengths, and the dried weights of the stems, leaves, and roots of the sugarcane stem cuttings when compared to the control treatment (T1) (Figure 9A–D,G). The highest values of the number of leaves were obtained for the sugarcane stem cuttings that were inoculated with each fungus along with the addition of Ca_3_(PO_4_)_2_ (T6–T8). However, the inoculation of *A. pseudopiperis* with the addition of Ca_3_(PO_4_)_2_ (T7) resulted in a greater root length compared to *A. chiangmaiensis* (T6) and *A. pseudotubingensis* (T8), while there was no statistically significant difference between these three treatments.

The results showed that the inoculation of each fungus with additional Ca_3_(PO_4_)_2_ (T6–T8) significantly increased the chlorophyll content in the leaves and the cellular inorganic phosphate content in the leaves, stems, and roots of the sugarcane stem cuttings when compared to the control treatment (T1), the addition of only Ca_3_(PO_4_)_2_ (T2), and the inoculation with each fungus (T3–T5) (Figure 9E,F).

## 3. Discussion

In the present study, three PSF, namely, *Aspergillus chiangmaiensis*, *A. pseudopiperis*, and *A. pseudotubingensis*, isolated from soils (loamy sand), were investigated for their plant-growth-promoting capabilities (the production of IAA, siderophores, and the solubilization of phosphorus minerals). IAA is a prevalent type of auxin found in plants and plays a key role in various plant growth reactions, such as elongation, division, the differentiation of cells, and the initiation of roots [34,35,36]. Several previous studies have found that soil fungi can produce IAA as a part of their metabolism, which plays a significant role in the growth and development of plants [32,33,37]. L-tryptophan (L-trp) served as the main precursor for the synthesis of IAA [38]. Several previous investigations have shown that PSF can produce IAA both with and without L-trp [22,32,39,40,41]. Our results revealed that *A. pseudopiperis* produced an average of 33.37 µg/mL of IAA when grown in a liquid medium supplemented with L-trp. This outcome is in accordance with previous studies that reported that less than 100 g/mL of IAA was produced from PSF in the genera, *Alternaria*, *Aspergillus*, *Chaetomium*, *Curvularia*, *Fusarium*, *Penicillium*, *Rhizopus*, and *Trichoderma*, isolated from soils [32,39,40,42,43,44,45,46]. Nevertheless, the quantity of IAA produced by PSF in the *Asperigllus* genus differed among various species and strains, with certain strains exhibiting higher levels of IAA production. For example, Li et al. [45] reported that *A. tubingensis* HZ123 could produce IAA at 267.38 µg/mL. Additionally, *A. tubingensis* SFSA1 and *A. fumigatus* SFSA7 showed the highest IAA productions at 260 and 212 µg/mL, respectively [46]. Moreover, numerous previous studies have demonstrated that the IAA production from PSF could improve and increase the root length in various plants [30,39,45,47,48,49].

Several fungi have demonstrated the ability to synthesize siderophores, which serve to chelate ferric iron in constrained environments. This process aids in the promotion of plant growth and enhances plant nutrition [50,51]. Siderophores also function as potential biocontrol agents against certain plant pathogens by decreasing the available iron from the host environment [52,53]. This study revealed that all strains of fungi exhibited a positive result for siderophore production. This outcome was consistent with prior studies that found that some species of PSF in the genera *Alternaria*, *Aspergillus, Curvularia*, *Fusarium*, *Mucor*, *Penicillium*, *Purpureocillium*, *Rhizopus*, *Talaromyces*, and *Trichoderma* could produce siderophores [33,40,44,45,48,54,55,56,57]. Additionally, siderophore production has been reported in *Aspergillus* species isolated from soils, including *A. aculeatus*, *A. brunneoviolaceus*, *A. niger*, *A. tubingensis*, and *A. violaceofuscus* [32,40,45,58,59].

The results indicated that three fungal species can solubilize phosphate in the forms of Ca_3_(PO_4_)_2_ and FePO_4_. However, all fungi were found to be unable to solubilize AlPO_4_. This result is supported by the findings of Hervieux et al. [60], Arriagada et al. [61], and Kolaei et al. [62], who reported that the growth of fungi is significantly inhibited by the presence of aluminum. In this study, *A. chiangmaiensis*, *A. pseudopiperis*, and *A. pseudotubingensis* could produce both acid and alkaline phosphatases and organic acids (oxalic acid, tartaric acid, and succinic acid) for the solubilizations of Ca_3_(PO_4_)_2_ and FePO_4_. These results are consistent with the numerous prior studies that have documented that mechanisms recognized to be responsible for the solubilization of phosphate minerals are the production of different types of organic acids as chelating substances, phosphatase enzymes, and other metabolites [3,14,63,64,65,66,67,68,69]. Moreover, several previous studies have reported that the capacity to solubilize phosphatases was contingent on the specific fungal genus, species, and strain [14,40,44,70,71]. Our results are corroborated by previous studies that demonstrated that PSF belonging to the genera *Aspergillus*, *Fusarium*, *Macrophomina*, *Penicillium*, *Talaromyces*, and *Trichoderma* produced phosphatases for solubilizing phosphate minerals [41,72,73,74,75]. Additionally, the ability to produce organic acids for solubilizing various forms of insoluble phosphorus sources relies on the specific fungal genus, species, and strain [41,76,77,78]. In this study, oxalic, tartaric, and succinic acids were identified in the liquid medium for solubilizing Ca_3_(PO_4_)_2_ and FePO_4_ by *A. chiangmaiensis*, *A. pseudopiperis*, and *A. pseudotubingensis*. Tartaric acid was dominant, followed by oxalic acid and succinic acid. This corresponds with the findings of Li et al. [79], who observed that *A. aculeatus* primarily produced tartaric acid for solubilizing Ca_3_(PO_4_)_2_, followed by citric and malic acids. According to Wang et al. [70], *A. niger* CS-1 could primarily produce oxalic acid during the solubilization of Ca_3_(PO_4_)_2_, which was then followed by tartaric acid and citric acid. Additionally, *A. niger*, *A. awamori*, *A. carbonarius*, and *A. tubingensis* could produce acetic, citric, formic, fumaric, gluconic, malic, propionic, succinic, tartaric, and oxalic acids in the solubilization of inorganic phosphate minerals [25,40,68,78,80,81,82].

In this study, all three fungi exhibited resistance to the abiotic conditions of drought, pH level, temperature, and salinity in the in vitro experiment. As a result, it was shown that these tolerances were influenced by the fungal species. The outcomes demonstrated that each fungal species had a high level of tolerance to drought, with water availability ranging from 0.837 to 0.859. In previous investigations, *A. aculeatus*, *A. fumigatus* SG-17, *A. violaceofuscus* MH220545, *Penicillium citrinum* 5TAKL-3a, *Pseudeurotium* sp. GRs12, *Talaromyces* sp. GS1, and *Trichoderma* GR21 have been found to be drought-tolerant in vitro, and their beneficial effects on plant growth and the enhancement of drought tolerance have been investigated in several plants, e.g., mulberry, perennial ryegrass, rice, tomato, and wheat [83,84,85,86,87]. All fungi were able to grow in both acidic and alkaline (pH 4–9) conditions. Therefore, these fungi could be cultivated in and endure typical soil conditions with pH levels between 6 and 8, as well as soils that are extremely acidic or alkaline. Our results were in accordance with the results of Rinu and Pandey [88], who reported that *A. candidus*, *A. deflectus*, *A. flavus*, *A. fumigatus*, *A. glaucus*, *A. nidulans*, *A. niger*, *A. parasiticus*, *A. sydowiihad*, and *A. wentii* could tolerate pH ranges from 2 to 12. The range of pH tolerance that *A. niger* and *A. japonicus* displayed was 4 to 11 [89]. In addition, *A. carbonarius* showed the highest growth at pH 4 to 6.5 [90]. In the present study, all three fungi were successfully grown at temperatures ranging from 20 to 40 °C. These results were similar to those of Passamani et al. [90], who found that *A. carbonarius* and *A. niger* could grow at temperatures from 20 to 37 °C. Kurniati et al. [91] indicated that *A. flavus* have readily grown between the temperatures of 25 and 42 °C. *Aspergillus niger* and *A. japonicus* exhibited levels of temperature tolerance ranging from 10 to 45 °C [89]. Furthermore, several *Aspergillus* species have a temperature tolerance ranging from 4 to 42 °C [88]. In this study, all fungi were tolerant to high concentrations of NaCl at 16 to 17%. The ability of the PSF to tolerate salt was demonstrated to depend on the fungal species and strain, as well as NaCl concentration. This result was consistent with the salt tolerance findings of Rinu and Pandey [88], who demonstrated that ten species of phosphate-solubilizing *Aspergillus* displayed varying degrees of salinity tolerance (at 12% up to 15% NaCl). Xiao et al. [89] reported that *A. niger* and *A. japonicus* were tolerant to salinity at up to 3.5% NaCl. Additionally, *A. niger*, *A. fumigatus*, *A. pulverulentus*, *A. parasiticus*, and *A. flavus* could tolerate NaCl concentrations of up to 9% [92]. Tolerance to various stresses is an important factor for the survival and growth of fungi, as is the capacity to solubilize insoluble minerals insoils [2,65,89,93]. Thus, the tolerance to drought, pH, temperature, and salinity of selected plant-growth-promoting microorganisms should be noted. These recommendations are considered to select and apply successful applications [94,95,96]. Subsequent studies ought to investigate the PSF obtained in this study for their potential to improve plant growth under conditions of drought, varying pH levels, temperature fluctuations, and salinity in both greenhouse and field experiments.

The agrochemical (herbicides, fungicides, and insecticides) tolerance of fungi varied depending on the type and concentration of agrochemicals, as well as the species and strain of fungi [97,98]. The growth and tolerance capacity of each fungal strain could be decreased by an increase in the agrochemical concentration, which was consistent with previous studies [99,100,101]. In this study, the fungicides (metalaxyl and benomyl) completely inhibited the mycelial growth of all fungi in all concentrations. Each fungal species exhibited varying degrees of tolerance to herbicides (glyphosate-isopropylammonium, 2,4-D-dimethylammonium, and paraquat dichloride) and insecticides (methomyl and propargite), which was contingent upon their species characteristics. It was found that *A. pseudopiperis* and *A. pseudotubingensis* could tolerate methomyl and propargite, respectively, in the field at recommended dosages. These findings are consistent with previous studies that demonstrated that some PSF species of the genera *Aspergillus*, *Chaetomium*, *Clonostachys, Grifola, Minimedusa, Mucor, Penicillium, Phycomyces, Pochonia, Purpureocillium, Rhizopus,* and *Trichoderma* could tolerate agrochemicals at various dosages depending on the species and strain [101,102,103,104,105,106]. Notably, Benito et al. [107] found that *A. flavus* AFS 63 and *A. parasiticus* APS 55 were able to tolerate glyphosate (herbicide) at the recommended field dosages. *Aspergillus oryzae* AM 1 exhibited the ability to tolerate chlorpyrifos (insecticide) at both the recommended field dosages and at over double the recommended dosages [108]. Therefore, the knowledge regarding the agrochemical tolerance of the PSF gained from this study could prove valuable insights for practical agricultural purposes, as it might involve the concurrent use of these fungal species with herbicides and insecticides on crops at recommended doses.

Microbial viability is an important feature when developing an inoculum as a biostimulant and plays a crucial role in successful commercialization [109,110]. The survival of microorganisms, carrier characteristics, biological effectiveness, and storage life of the biostimulant inoculum are affected by a variety of factors, including the drying process [111,112] and various storage conditions (e.g., moisture content, temperature, and sunlight intensity) [113,114,115]. Several researchers have investigated the effect of various temperatures on the shelf life of different microbial inocula [116,117,118]. Previous studies mentioned that maintaining the viability of microbes that are present in microbial inoculant formulations is challenging, and a loss of viability of less than 50% was considered to be non-effective for application [119,120]. Our results found that the viability of all fungi in a granular inoculum was higher than 50% after storage for 3 months at 4 and 20 °C. The viability of *A. chiangmaiensis* was higher than 50% after storage at 4–37 °C for 4 months, while the viability of *A. pseudopiperis* was higher than 50% at 20 °C after 4 months of storage. Furthermore, the viability of *A. pseudotubingensis* was higher than 50% after storage at 4–37 °C for 3 months. The rapid decrease in viability (<50%) of all fungal species in the granular inoculum was found at high temperatures (45–50 °C). Our findings are corroborated by previous research that found that the survival ability of the microbial species in inoculum depends on the species and strain, a suitable carrier, and the storage temperature [114,118]. Low temperatures are generally recommended for inoculum storage for prolonged microbial viability and high-quality products [49,114,121]. However, the inoculum has a long microbial viability at room temperature, which offers a financial advantage and provides more convenience to users [110,122,123]. The high temperatures have effects on the growth of microbes and decrease conidia germination due to changes in the membrane structure and protein degradation [118]. Prior to this study, Accinelli et al. [124] reported that the viability of *A. flavus* NRRL 30797 on Mater-Bi granules showed a greater decline at 25 °C than at 5 °C during the storage period of six months. The phosphate-solubilizing fungus, *A. awamori* S29, had a viability of 56.4% after six months of storage at room temperature (28 °C) in a free-form-based bio-formulation [125]. A study conducted by Wang et al. [49] indicated that the viability of the phosphate-solubilizing fungus, *A. niger* 1107, in a biofertilizer was more than 50% when stored at 4 °C for up to seven months. Therefore, storage conditions are most important in maintaining the steadiness and quality of an inoculum. 

In the current study, the results demonstrated that inoculation stem cuttings of sugarcane and cassava with the granular inocula of *A. chiangmaiensis*, *A. pseudopiperis*, and *A. pseudotubingensis* could significantly increase root induction and plant growth. Moreover, the inoculation of each fungus and the simultaneous addition of an insoluble mineral phosphate, Ca_3_(PO_4_)_2_, considerably enhanced the root induction and growth of the sugarcane and cassava stem cuttings. Our results are supported by previous studies that showed that PSF, including species of *Aspergillus* spp., have the ability to dissolve insoluble mineral phosphates in soil, thereby elevating the concentration of accessible phosphorus for enhanced plant growth and productivity [2,3,20,126]. Tariq et al. [127] reported that the inoculation of PSF, *A. versicolor* MF, could significantly increase the root lengths of mint plants (*Mentha viridis*). In greenhouse experiments, the application of PSF, including *A. awamori* S29, *A. awamori* S-19, *A. niger* 1107, *A. niger* FS1, and *A. tubingensis* QF05, along with the addition of an insoluble mineral phosphate, resulted in the enhanced root development and growth of Chinese cabbage (*Brassica rapa*), chickpea (*Cicer arietinum* L.), mung bean (*Vigna radiata*), tomato (*Solanum lycopersicum* L.), and white clover (*Trifolium repens*) [25,30,32,49,126]. Additionally, in the experiments conducted by Kaur and Reddy [27], it was found that the inoculation of PSF, in the form of *A. niger* PSF-7 and *A. tubingensis* PSF-4, could significantly enhance the root lengths, plant growths, and yields of maize (*Zea mays* L.) and wheat (*Triticum aestivum*) in a field experiment. The results of our research revealed that both the sugarcane and cassava stem cuttings had increased chlorophyll and cellular inorganic phosphate concentrations following the inoculation of each fungal strain. Our findings are consistent with previous reports demonstrating that the inoculation of PSF, in the form of *A. awamori* S-19, *A. flavus*, and *A. niger* CSR3, led to a significant increase in the chlorophyll levels of chickpea, maize, and tomato [30,128,129]. Peng et al. [130] reported that the inoculation of six strains of *A. niger* resulted in an increase in the root lengths, plant growths, and chlorophyll contents in peanut plants (*Arachis hypogaea* L.). The phosphorus contents of chickpea, Chinese mustard (*Brassica chinensis* Linn.), and soybeans (*Glycine max*) could be increased via PSF inoculation, including *A. awamori* VHI + VQ2, *A. niger* 6A, and *A. niger* K7 [22,29,131]. Moreover, Shahrajabian et al. [132,133] documented that the application of biostimulants not only has a beneficial effect on plant growth and productivity, but it may also lead to environmentally friendly practices and increase the use efficiency of natural resources for the sustainability of agricultural and horticultural production systems.

## 4. Materials and Methods

### 4.1. Fungal Strains

Three phosphate-solubilizing fungi, namely *A. chiangmaiensis* SDBR-CMUI4, *A. pseudopiperis* SDBR-CMUI1, and *A. pseudotubingensis* SDBR-CMUO2, were isolated from the soil in a longan orchard [23]. All strains of fungi were preserved in the culture collection of the Sustainable Development of Biological Resources, located at the Faculty of Science, Chiang Mai University in Chiang Mai Province, Thailand. The fungi were grown on potato dextrose agar (PDA; Conda, Madrid, Spain) and kept in an incubator set at 25 °C.

### 4.2. Characterization of Plant Growth Promotion Properties

#### 4.2.1. Determination of IAA Production

All fungal strains were cultivated on PDA at a temperature of 30 °C for one week before being used. Five mycelial plugs (5 mm in diameter) taken from the edge of a colony were inoculated into a 125 mL Erlenmeyer flask containing 30 mL of potato dextrose broth (PDB; Conda, Madrid, Spain), with a pH level of 6.0, added to a concentration of 0.2 mg/mL of L-tryptophan (L-trp; Sigma-Aldrich, Steinheim, Germany). The inoculation was carried out in the dark at 25 °C using a reciprocating shaker at 150 rpm. The cultures were centrifuged at 11,000 rpm for 15 min to collect the supernatant after seven days of incubation. The production of IAA was initially assessed using a colorimetric assay by using Salkowski’s reagent, following the method described by Tsavkelova et al. [134]. A positive result for IAA production was indicated by a pink to red color. Three replications were made for each treatment. According to the procedure outlined by Kumla et al. [135], the IAA present in the supernatant was extracted and confirmed using high-performance liquid chromatography (HPLC).

#### 4.2.2. Determination of Siderophore Production

Chrome Azurol S (CAS) agar was used to evaluate siderophore production [136]. Colonies were grown in PDA at 30 °C for 7 days; then, mycelial plugs (5 mm in diameter) were taken out from those colonies and placed on CAS agar. The inoculation plates were incubated for three days at 30 °C in the dark. Fungal strains that exhibited a yellow, orange, purplish-red, or purple zone around their colonies were classified as strains capable of producing siderophores [137]. Three replications were set up for each treatment.

#### 4.2.3. Solubilization of Phosphate Minerals, Phosphatase Activities, and Organic Acid Production

Quantitative estimation of soluble phosphate mineral solubilization was performed using basal liquid media (10.0 g glucose, 0.5 g (NH_4_)_2_SO_4_, 0.2 g NaCl, 0.1 g MgSO_4_·7H_2_O, 0.2 g KCl, 0.5 g yeast extract, 0.002 g MnSO_4_·H_2_O, and 1000 mL of deionized water; pH 7.0) with the addition of AlPO_4_, Ca_3_(PO_4_)_2_, and FePO_4_ to the desired final concentration of 0.5% (*w*/*v*), according to the procedure outlined by Fomina et al. [138]. The starting pH of the medium was set to 7.0 prior to sterilization. Mycelial inocula were formed through culturing on PDA at 30 °C in complete darkness for a period of seven days. Mycelial plugs (with a diameter of 5 mm) taken from the outer edge of the developing colony were utilized for inoculation into the broth media. The control treatment (uninoculated treatment) was run with each experiment without fungi. Three replications were made for each treatment. The flasks were subsequently incubated at 25 ± 2 °C with shaking at 125 rpm. The remaining available phosphorus in the culture supernatant was assessed at intervals of three days over a period of 15 days. The cultures were passed through Whatman number 1 filter paper and then subjected to centrifugation at a speed of 11,000 rpm for a duration of 10 min in order to eliminate any suspended particles and fragments of mycelium. The obtained culture supernatants were used for the determination of pH value, available phosphorus concentration, and phosphatase activities, as well as organic acid production.

A pH meter was used to test the pH of the culture supernatants [139]. Subsequently, the molybdenum blue technique was used to determine the available phosphorus concentration [140,141]. The 500 µL culture supernatant was combined with an equal volume of 10% (*w*/*v*) trichloroacetic acid in a test tube; then, 4 mL of a color reagent (consisting of a 1:1:1:2 ratio of 6 N H_2_SO_4_/2.5% (*w/v*) ammonium molybdate/10% (*w/v*) ascorbic acid/distilled water) was added. The mixture was left to incubate at room temperature (25 ± 2 °C) for 15 min. An uninoculated medium was used as the blank. A spectrophotometer was used to measure the absorbance of the developing blue color at 820 nm. The concentration of available phosphorus was calculated from a calibration curve of KH_2_PO_4_, and the data are expressed as milligrams per liter (mg/L).

The supernatant of each fungal culture from the previous experiment was determined for phosphatase activities. Acid and alkaline phosphatase assays were performed according to the procedures outlined by Gaind and Singh [142] and Adhikari and Pandey [143], respectively. For the acid phosphatase assay, the culture supernatant (0.4 mL) was mixed with 1 mL of 0.1 M citrate buffer (pH 5.0), 0.5 mL of 0.05 M *p*-nitrophenol phosphate (*p*NPP), and 0.1 mL of 1 mM MgCl_2_. The alkaline phosphatase assay was performed using the same method, but 0.1 M glycine–sodium hydroxide buffer (pH 9.0) was used in place of the citrate buffer. The reaction mixture was incubated at 37 °C in darkness for 60 min. One hundred microliters of 5 M NaOH was added to stop the process. An uninoculated medium was used as the blank. A microplate spectrophotometer was used to measure the intensity of the produced yellow color at 400 nm. The quantity of liberated *p*-nitrophenol (*p*NP) was measured using a *p*-nitrophenol standard as a reference. The phosphatase activity is expressed as 1 µmol *p*NP/mL/h.

The organic acids in the culture supernatant from the broth liquid medium supplemented with different sources of Ca_3_(PO_4_)_2_ and FePO_4_ were analyzed via HPLC. The culture supernatant was filtered using a 0.45 m syringe filter (MILLEX^®^HA, Merck Millipore Ltd., Tullagreen, Carrigtwohill, County Cork, Ireland) with a volume of one milliliter. The organic acids in the filtrate samples were analyzed using a Shimadzu VP series HPLC system equipped with an SPD-10AVP UV/VIS detector (Shimadzu, Europe, Duisburg, Germany). The temperature was adjusted to 40 °C and the column was an Ultra-Aqueous C18 column (250 × 4.6 mm, 5 µm; Restek Corporation, Benner Circle Bellefonte, PA, USA). The mobile phase included 0.1% phosphoric acid solution flowing at 0.8 µL/min, with a sample volume of 2 µL for each injection. The detection wavelength was 214 nm. A calibration curve was established using five organic acid standards containing varying levels of citric, malic, oxalic, succinic, and tartaric acids. The quantification of organic acids was achieved by comparing the peak areas in the supernatant samples with those on the calibration curve. The results are presented as micrograms of organic acid per milliliter (µg/mL).

### 4.3. Drought, pH, Temperature, and Salinity Tolerances

#### 4.3.1. Drought Tolerance

The technique outlined by Hallsworth et al. [144] was used to assess each fungal strain’s resistance to drought using PDA supplemented with sorbitol powder. The sorbitol concentrations were set at 0, 85, 175, 285, 405, 520, 605, 660, and 780 g/L, corresponding to the water activity (a_w_) levels of 0.998, 0.995, 0.993, 0.963, 0.930, 0.912, 0.886, 0.859, and 0.837, respectively. Three replications were made for each fungal strain.

#### 4.3.2. pH Tolerance

The pH tolerance was determined using PDA. The pH of the medium was modified to the values of 3, 4, 5, 6, 7, 8, and 9 by adding either 1 N HCl or 1 N NaOH before being autoclaved. Mycelial plugs (5 mm in diameter) were placed on the test media. The plates were then incubated at 30 °C after being inoculated. Fungal growth in the tested media was monitored five days later. Three replications were made for each fungal strain.

#### 4.3.3. Temperature Tolerance

PDA was employed to conduct this experiment. A mycelial plug of each fungal strain was inoculated on PDA and incubated at 0, 4, 10, 15, 20, 25, 30, 35, 40, and 45 °C in the dark. After five days of incubation, the growth of fungi in the tested media was measured. All experiments were performed in three replicates.

#### 4.3.4. Salinity Tolerance

The salinity tolerance was determined using PDA supplemented with a graded series of NaCl concentrations (0, 5, 10, 15, 16, 17, 18, 19, and 20%) according to the methods described by Rinu and Pandey [88] and Tresner and Hayes [145]. The pH of all of the tested media was modified using either 1 N HCl or 1 N NaOH before being autoclaved. Five-millimeter-diameter mycelial plugs were placed in the center of the test medium and incubated at 30 °C in the dark. After five days of incubation, the fungal growth of the fungi was observed. Three replications were carried out for each treatment.

### 4.4. Agrochemical Tolerance

Three commercially available herbicides, including glyphosate-isopropylammonium (Glyphosate 48^®^; Pareto Agro Co., Ltd., Bangkok, Thailand), 2,4-D-dimethylammonium (DMA6^®^; Zagro (Thailand) Ltd., Pathumthani, Thailand), and paraquat dichloride (Grammoxone^®^; Syngenta Crop Protection Co., Ltd., Bangkok, Thailand); two commercial fungicides, including metalaxyl (Lonsan^®^; Sahaphol Kemekaset Ltd., Part., Bangkok, Thailand) and benomyl (Belly OD^®^; Sahaphol Kemekaset Ltd., Part., Bangkok, Thailand); and two commercial insecticides, including methomyl (Garnet^®^; Alpha Agro Tech Co., Ltd., Samut Prakan, Thailand) and propargite (Omite-20^®^; Sotus International Co., Ltd., Nonthaburi, Thailand), were tested in this experiment following the methodology outlined by Suwannarach et al. [99]. The recommended dosage for field applications of glyphosate-isopropylammonium, 2,4-D-dimethylammonium, paraquat dichloride, metalaxyl, benomyl, methomyl, and propargite were 7000, 2500, 6250, 2000, 1500, 1750, and 2000 ppm, respectively. Each compound was prepared and introduced into autoclaved PDA in order to achieve final concentrations ranging from half of the recommended dosage to quadruple the recommended dosage. A sterile cellophane disc was used to cover the surface of the test media, and the fungal mycelial plug (with a diameter of 5 mm) was then positioned on the test media [135]. The plates were incubated in the dark at 25 °C. Following a five-day incubation period, the cellophane disc was taken off and subjected to drying at 60 °C for 48 h. Mycelium dry weights were then calculated. The formula provided by Fomina et al. [138] was used to determine the tolerance Index (TI). TI values of 0% indicated a lethal outcome, while values below 50% signified a growth-inhibiting effect. Three replications of each treatment were performed.

### 4.5. Evaluation of Fungal Viability in a Granular Inoculum

#### 4.5.1. Preparation of Granular Inoculum

The fungal strain was cultured on PDA for one week at a temperature of 37 °C. A Petri plate containing 5 mL of sterile deionized water was gently scraped to collect conidia. The concentration of conidia in the suspension was determined using a hemocytometer observed through a microscope. Vermiculite, perlite, and peat moss were combined together to produce the carrier material in a ratio of 5:2:3 (*w/w/w*). The combined carrier material was subjected to a drying process at 70 °C for 72 h, and then processed through a blender, filtered using a 2 mm mesh, and finally, sterilized twice in an autoclave set at 121 °C for 30 min. The conidial suspension of each fungus was combined with the sterilized carrier material to achieve a final concentration of approximately 1 × 10^7^ conidia/g [110]. A tablet pressure machine was used in the granulation process. Subsequently, the granules were dried in an oven at 45 °C for 48 h. Each granular inoculum used in this experiment had an overall diameter of 36 mm and 10 mm length.

#### 4.5.2. Evaluation of Fungal Viability

The viability of fungal conidia in a granular inoculum was investigated. Ten grams of granular inoculum product were packed in 4 oz (4.5 × 9.0 cm) sterilized glass bottles with screwable plastic lids and stored in the dark at different storage temperatures including 4, 20, 25, 25 ± 2 (room temperature), 30, 37, 45, and 50 °C for five months. Three replicates were carried out for each temperature. The fungal viability was monitored at monthly intervals. The viable count was evaluated using the serial dilution spread plate method [146]. A total of 9 mL of the sterile saline solution (0.5% NaCl, *w/v*) was used to suspend 1 g of granules. Suspensions were vigorously shaken using a vortex mixer for 1 min; then, serial dilutions were made. After that, 0.1 mL of each serial dilution was dropped onto PDA and spread. The plates were kept at 30 °C for three days while being kept in the dark. The total number of fungal colonies were counted and recorded. The shelf life of biofertilizer product was calculated and expressed as a percentage (%).

### 4.6. Root Inductions of Cassava and Sugarcane Stem Cuttings under Greenhouse Conditions

The cassava (*M. esculenta* Crantz) cultivar, “Kaeg Dum”, and sugarcane (*S. officinarum* L.) cultivar, “Khon Kaen”, were used in this experiment. This experiment employed soil from sugarcane and cassava fields with a pH range of 6.8 to 6.9 as the planting medium. Sterilization of the soil was performed twice at 121 °C for 30 min each. A completely random design (CRD) was used to set up this experiment. The details of each treatment employed in this study are listed in Table 2. The 15 cm long stem cuttings for planting were obtained from the stakes of 10-month-old plants. The stem cuttings were transferred into each plastic pot (19.5 × 13 × 14.5 cm) containing 3 kg of soil in each experiment. Ten replications of each treatment were run twice. The plants were cultivated for a duration of 60 days in a greenhouse situated at the Faculty of Science at Chiang Mai University in Chiang Mai Province, Thailand between September and October 2022. The greenhouse experienced temperature fluctuated between 25 and 35 °C and relative humidity levels ranging from 50% to 85%. The daily peak light intensity varied within the range of 16,000 to 25,000 lux.

#### 4.6.1. Measurement of Plant Growth

The heights of the plants, total number of leaves, leaf lengths, main root lengths, and dry weights of the roots and leaves were measured and recorded for both cassava and sugarcane plants.

#### 4.6.2. Determination of Chlorophyll and Cellular Inorganic Phosphate Contents in Plants

The methodologies outlined in the studies by Lichtenthaler and Wellburn [147] and Liang et al. [148] were used to determine the concentrations of chlorophyll (chlorophyll a, chlorophyll b, and total chlorophyll) in the leaves of the cassava and sugarcane plants. A total of 0.2 g of fresh leaf samples was submerged in 8 mL of an 80% (*v/v*) acetone solution, followed by incubation for 24 h at 25 °C in darkness until the tissue became white. A microplate spectrophotometer was employed to measure the supernatant at 645 and 663 nm. Chlorophyll concentration was computed, and the results were represented in milligrams per gram (mg/g).

The methodology outlined by Ames [149] and Wang et al. [150] was used to measure the concentrations of cellular inorganic phosphate. Plant tissues were weighed and soaked in 1 mL of 1% glacial acetate. Eight occurrences of freezing and thawing followed. Subsequently, 100 µL of the extract was combined with 700 µL of a phosphate reaction buffer (consisting of A = 0.42% ammonium molybdate and 2.85% (*v/v*) sulfuric acid and B = 10% (*w/v*) ascorbic acid; A/B (*v*/*v*) in a ratio of 6:1) and 200 µL of deionized water. A spectrophotometer was used to determine the absorbance at 820 nm after the reaction had been carried out at 37 °C for 60 min. A dipotassium hydrogen phosphate calibration curve was used to determine the concentrations of cellular inorganic phosphate, and the results are expressed as µmol/g fresh weight (µmol/g FW).

### 4.7. Statistical Analysis

Statistical variances among the treatments were evaluated using one-way analysis of variance (ANOVA) conducted with the SPSS software for Microsoft Windows (version 16). Significant differences at *p* ≤ 0.05 were determined using Duncan’s multiple range test (DMRT).

## 5. Conclusions

In the present study, three phosphate-solubilizing *Aspergillus* species, *A*. chiangmaiensis, *A. pseudopiperis*, and *A. pseudotubingensis,* could produce siderophores and solubilize the insoluble mineral phosphate minerals (Ca_3_(PO_4_)_2_ and FePO_4_). These three fungal species produce both acid and alkaline phosphatases and organic acids (tartaric, oxalic, and succinic acids) that indicate the mechanisms for solubilizing insoluble phosphate minerals. Moreover, *A. pseudopiperis* could produce IAA. Furthermore, their tolerances to drought, pH value, temperature, salinity, and agrochemicals as well as their fungal viability were characterized for consideration in the field applications. The application of a granular inoculum of each fungal strain as a biostimulant to the cassava and sugarcane stem cuttings enhanced root development and did not cause any disease symptoms. Moreover, the presence of these three fungi along with the addition of Ca_3_(PO_4_)_2_ led to significant enhancements in the growths, chlorophyll contents, and cellular inorganic phosphate levels in both the cassava and sugarcane plants. Thus, these three fungi have the potential to serve as biostimulants to promote plant growth. Subsequent research will concentrate on utilizing the biostimulants derived from these three fungi to assess their impacts on the growths and yields of various plant species through field experiments. This endeavor will provide researchers with improved insights into the development of efficient biostimulants that have the potential to replace the detrimental chemical-based fertilizers presently employed in agriculture. To establish the safety of these three fungi, clinical investigations on toxicity will be necessary in further studies.

## Figures and Tables

**Table 1:** Plant growth promotion properties and tolerance of fungi in this study.

Plant Growth Promotion Properties	*A. chiangmaiensis* SDBR-CMUI4	*A. pseudopiperis* SDBR-CMUI1	*A. pseudotubingensis* SDBR-CMUO2
IAA production (µg/mL)	−	33.37	−
Siderophore production	+	+	+
Drought tolerance (a_w_)	0.837–0.998	0.859–0.998	0.837–0.998
pH tolerance	4.0–9.0	4.0–9.0	4.0–9.0
Temperature tolerance (°C)	4–40	4–40	4–40
Salinity tolerance (% NaCl)	Up to 17%	Up to 16%	Up to 17%

“+”: positive result; “−”: negative result.

**Table 2:** Details of the treatments in this study.

Treatment Number	Treatment Details
T1	Soil (control)
T2	Soil (3 kg) + Ca_3_(PO_4_)_2_ (1.5 g)
T3	Soil (3 kg) + inoculum of *Aspergillus chiangmaiensis* SDBR-CMUI4 (3 g)
T4	Soil (3 kg) + inoculum of *Aspergillus pseudopiperis* SDBR-CMUI1 (3 g)
T5	Soil (3 kg) + inoculum of *Aspergillus pseudotubingensis* SDBR-CMUO2 (3 g)
T6	Soil (3 kg) + Ca_3_(PO_4_)_2_ (1.5 g) + inoculum of *A. chiangmaiensis* SDBR-CMUI4 (3 g)
T7	Soil (3 kg) + Ca_3_(PO_4_)_2_ (1.5 g) + inoculum of *A. pseudopiperis* SDBR-CMUI1 (3 g)
T8	Soil (3 kg) + Ca_3_(PO_4_)_2_ (1.5 g) + inoculum of *A. pseudotubingensis* SDBR-CMUO2 (3 g)

## Data Availability

Not applicable.
